# Survey on perioperative tranexamic acid use

**DOI:** 10.1111/bjh.70529

**Published:** 2026-05-10

**Authors:** Louise H. Strickland, Hayley G. Evans, Olivia C. Robinson, Samantha Warnakulasuriya, Robbie Foy, Simon J. Stanworth

**Affiliations:** ^1^ Oxford University Hospitals Foundation Trust Oxford UK; ^2^ Nuffield Department of Orthopaedics, Rheumatology and Musculoskeletal Sciences (NDORMS) University of Oxford Oxford UK; ^3^ NIHR Blood and Transplant Research Unit in Data Driven Transfusion Practice, Nuffield Division of Clinical Laboratory Sciences, Radcliffe Department of Medicine University of Oxford Oxford UK; ^4^ Leeds Institute of Health Sciences University of Leeds Leeds UK; ^5^ Department of Anaesthesia and Perioperative Medicine University College London Hospital NHS Foundation Trust London UK

**Keywords:** major blood loss, surgical practice, tranexamic acid

To the Editor,

Tranexamic acid (TXA) is a safe and effective component of perioperative patient blood management, yet its use across surgical specialities remains variable. Despite strong evidence of benefit,^1^ clinicians differ in their assessment of risks, indications and routine use. To test whether behavioural influences identified in a single‐centre interview study^2^ generalise more widely, we undertook a national survey of clinicians involved in perioperative care.

An online questionnaire was distributed through UK perioperative professional networks, including the Perioperative Quality Improvement Programme (PQIP). Eligible participants were National Health Service (NHS) clinicians with perioperative responsibilities. The survey was based on the Theoretical Domains Framework (TDF),^3^, ^4^ capturing cognitive, emotional, social and environmental determinants of clinical behaviour. One item addressed each of 11 domains using 1–5 Likert scales, with optional free‐text comments (Figure S1). The survey was piloted among representative clinicians. This work was registered as a service evaluation by the sponsor National Health Service Blood & Transplant (NHSBT) and did not require additional NHS research ethics approval. Invitations were distributed through peri‐operative professional networks, reaching approximately 4200 clinicians. The survey was open from 24 January 2025 to 8 May 2025, and 106 responses were received (estimated response rate 2.5%). No financial incentives were offered but a certificate was available as evidence for Continuing Professional Development (CPD). Mean ratings were summarised and visualised using heat maps for all respondents and for anaesthetists and surgeons separately. Free‐text responses were coded deductively to the TDF by two researchers with consensus resolution.

A total of 106 clinicians from 32 hospitals responded: 77 anaesthetists (72.6%), 28 surgeons (26.5%) and one surgical care practitioner (0.9%) (Table 1; Figures S2 and S3). Respondents rated beliefs about TXA benefits and their own knowledge, skills and confidence as the strongest influences on use. Role clarity, decision‐making support and motivation were rated moderately influential. Environmental, social and behavioural domains generally received lower mean ratings, although several, including behavioural regulation, showed marked variation across respondents (Figure 1). The heat maps show the number of respondents rating the level of importance attributed to each domain in the use of TXA from not important at all through to extremely important. Higher ratings correspond to ‘hot’ (red) and lower ratings correspond to ‘cold’ (blue). The highest scoring domains in terms of being very/extremely important with the highest number of responses included knowledge and skills (*n* = 91), professional roles and identities (*n* = 78), beliefs about capabilities (*n* = 78) and beliefs about consequences (*n* = 88). The domains which participants ranked lowest in terms of importance (not important at all or only slightly important) to using TXA were behavioural regulation (*n* = 66) and nature of the behaviour (*n* = 66). Some domains, such as memory and attention, showed higher ratings of importance among anaesthetists (*n* = 51) than surgeons, reflecting differences in workflow and decision processes. Sixty‐three respondents provided free‐text comments. These closely reflected themes from the earlier qualitative study^2^ and included variation in perceived thrombotic risk, differences in local practice norms and uncertainty in borderline cases (Table S1). Several respondents reported inconsistent protocols and ambiguity about responsibility for TXA prescribing.

**TABLE 1:** Tranexamic acid (TXA) survey participant demographics.

Profession	Consultant	Non‐consultant doctor	Other
Anaesthetist	67	10	
Surgeon	24	4	
Surgical care practitioner			1

*Note*: Survey participants (*n* = 106).

**FIGURE 1:**
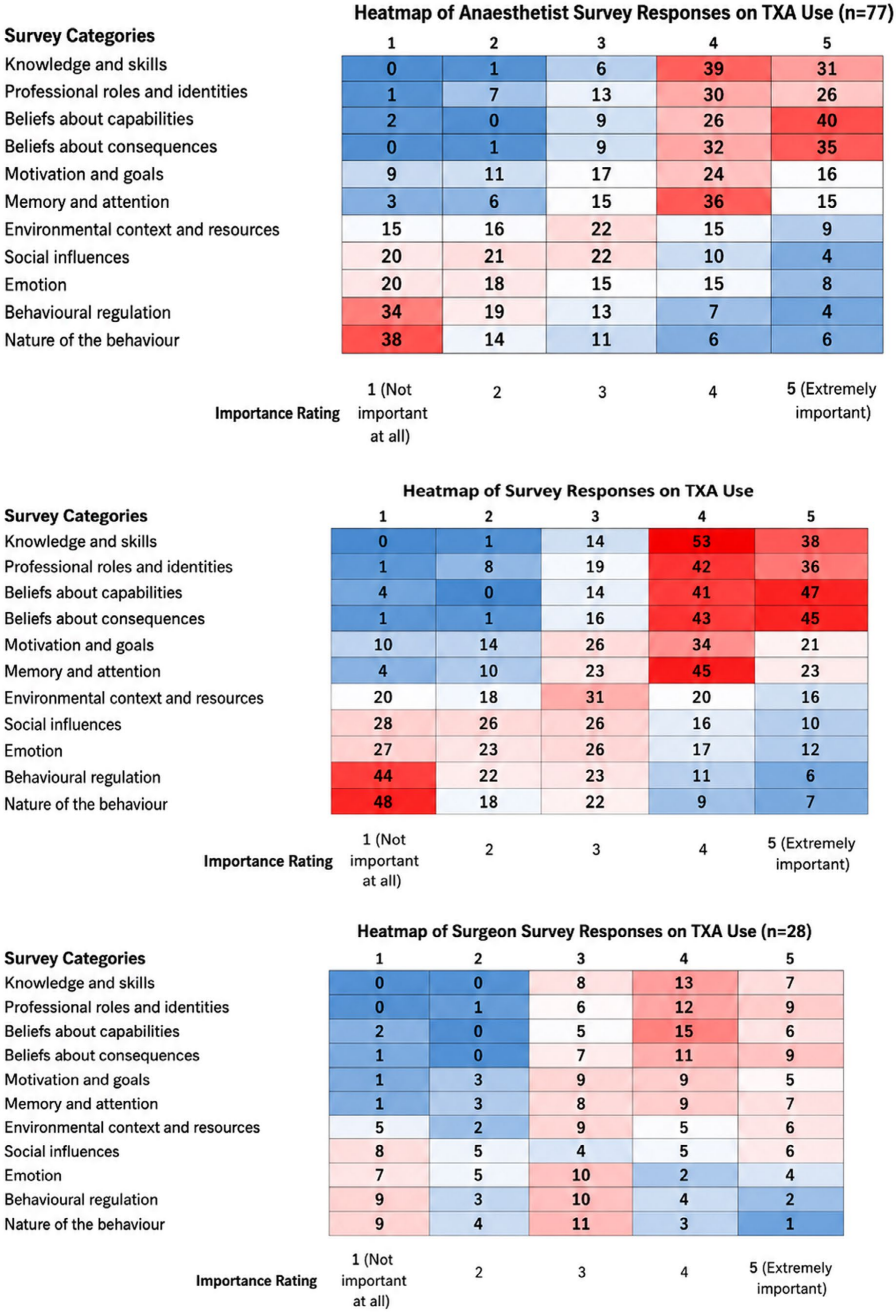
Tranexamic acid (TXA) survey heat maps. [Colour figure can be viewed at wileyonlinelibrary.com]

Survey findings corroborate and extend the behavioural influences identified previously and these findings are further supported by free‐text comments provided by respondents. The findings suggest two priorities for implementation: (1) strengthening clinicians' knowledge and confidence through targeted education and audit feedback^5^ and (2) clarifying roles and decision pathways, for example, through TXA prompts within World Health Organization (WHO) checklists or explicit prescribing responsibilities. Such actions could be integrated within routine PQIPs.

Limitations should be acknowledged. This was a self‐selected sample with a low response rate (~2.5%), reflecting dissemination through open professional networks. Our results may not represent surgical opinion overall but still offer insights to guide implementation activities. The majority of respondents were anaesthetists, reflecting the survey population, which may skew findings towards their perspectives. Our single‐item TDF questions were intentionally pragmatic but cannot provide the measurement reliability or detail that a more extensive survey might have offered. Survey findings will inform strategies to promote more consistent evidence‐based use of TXA, which should be considered in further quality improvement initiatives in surgery.

What this study adds:

- Influences on perioperative TXA use identified in a single‐site qualitative study were largely confirmed in a national survey.
- While environmental and social influences were rated as relatively less important, free‐text responses revealed meaningful influences related to team culture, local norms and role ambiguity.
- Priorities for implementation include strengthening clinician knowledge and confidence and clarifying decision roles and pathways for TXA use within perioperative workflows.

## AUTHOR CONTRIBUTIONS

**Samantha Warnakulasuriya:** Writing – review and editing; data curation. **Louise H. Strickland:** Methodology; formal analysis; data curation; writing – review and editing; writing – original draft; project administration. **Simon J. Stanworth:** Conceptualization; methodology; writing – review and editing; writing – original draft; data curation; funding acquisition; investigation. **Robbie Foy:** Conceptualization; writing – original draft; writing – review and editing; methodology; data curation. **Olivia C. Robinson:** Writing – review and editing; formal analysis; data curation. **Hayley G. Evans:** Methodology; data curation; formal analysis; project administration; writing – original draft; writing – review and editing.

## FUNDING INFORMATION

This publication is supported by the National Institute for Health and Care Research (NIHR) Blood and Transplant Research Unit in Data Driven Transfusion Practice (NIHR203334).

## CONFLICT OF INTEREST STATEMENT

The authors have no conflicts of interest to disclose.

## ETHICS STATEMENT

This work was registered and approved as a service evaluation by the NHSBT.

## Supporting information


**Figure S1.** Survey questions and instructions.

**Figure S2.** Overall survey specialities.

**Figure S3.** Survey geography.

**Table S1.** TXA use in surgery interview and survey data.

## Data Availability

Data are available from the corresponding author upon reasonable request.
