# Supplementary figures and images for: Survey on perioperative tranexamic acid use
Source: Br J Haematol. 2026 May 10;209(1):363–5. doi: 10.1111/bjh.70529 (PMC13340493; doi:10.1111/bjh.70529)

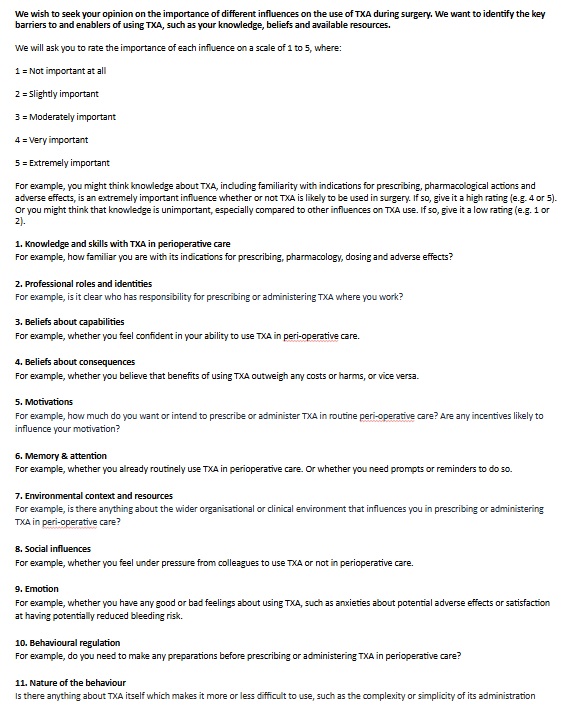

Supplement: Supplementary file 1 — Figure S1. Survey questions and instructions. [file BJH-209-363-s004.jpg]

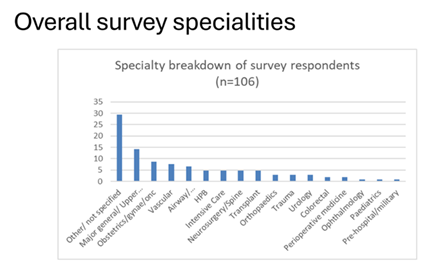

Supplement: Supplementary file 2 — Figure S2. Overall survey specialities. [file BJH-209-363-s001.png]

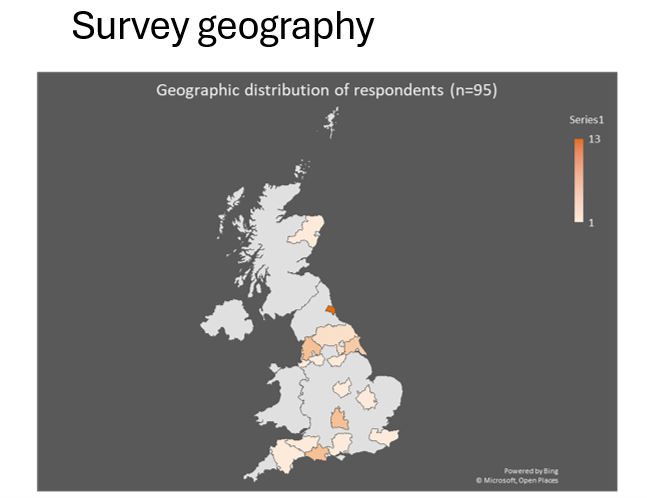

Supplement: Supplementary file 3 — Figure S3. Survey geography. [file BJH-209-363-s002.png]
