# Supplementary material for: Survey on perioperative tranexamic acid use
Source: Br J Haematol. 2026 May 10;209(1):363–5. doi: 10.1111/bjh.70529 (PMC13340493; doi:10.1111/bjh.70529)
Supplement: Supplementary file 4 — Table S1. TXA use in surgery interview and survey data. [file BJH-209-363-s003.docx]

Supplementary Table 1: TXA use in surgery interview and survey data

| **Domain** | **Summary of domain** | **Influence question area** | **Interview or Survey data** | **Example Quotes** | **No. of transcripts** | **Richness of description** |  |
| --- | --- | --- | --- | --- | --- | --- | --- |
| **Capability** |  |  |  |  |  |  |  |
| **1. Behavioural regulation** | *Goal/target setting, moderators of intention,*  *action planning and self-monitoring* | Planned usage of TXA to presurgical patients | Interview | I use it on every single patient that I'm expecting major blood loss (Pt1, Q1) | (n=21) | High |  |
|  |  |  | Interview | And most of the vascular surgeons do not administer TXA, even though, you know, we're ticking dutifully on the form that there is a risk of more than 500 mils blood loss (Pt10, Q30). |  |  |  |
|  |  |  | Survey | I use TXA in all spine operations irrespective of the preop estimated blood loss |  |  |  |
|  |  |  | Survey | I think there is some inertia around starting to use txa more |  |  |  |
|  |  |  | Survey | Nil - protocoled use in our department for arthroplasty. |  |  |  |
|  |  |  | Survey | As anaesthetic group we always use TXA. We give it for surgeries expecting more bleeding or if we see the surgical site is oozy or in Major haemorrhage scenario. The cut off of 500 ml might be low. We rather give it for more then a little expected blood loss |  |  |  |
|  |  |  | Survey | We almost routinely use TXA where appropriate and it is discussed at briefs for both elective and emergency surgery as far as I am aware. |  |  |  |
|  |  |  | Survey | Obstetric anaesthetists tend to use it more |  |  |  |
|  |  |  | Survey | Routine part of major orthopaedic work. Commonly used in urology/gynae/general surgical patients. |  |  |  |
| **2. Knowledge** | Knowledge about topic,  scientific rationale for using and procedural knowledge | Clinicians understanding of how TXA works | Interview | I’ve attended many anaesthesia conferences, many regular anaesthesia conferences over the last sort of three to five years and TXA has routinely come up in these conferences. Explaining the benefits of TXA (Pt22, Q55). | (n=18) | High |  |
|  |  |  | Interview | You know it’s just a drug something to do with clotting. There's a there's a sort of a misunderstanding that it forms clots and enables clots to form, which isn't what it does at all (Pt5, Q15). |  |  |  |
|  |  |  | Survey | 1) Widespread surgical ignorance of benefits vs risks, and 2) almost surgical arrogance that the proposed surgical technique will not, in their hands, possibly result in a blood loss of >500mls |  |  |  |
|  |  |  | Survey | There are many factors influencing bleeding tendency, and I usually know within the first few minutes how the patient will behave. This often determines my TXA prescription. Despite doing very major 10-12h+ cancer resections we very rarely lose more than a few hundred mls, almost never need a transfusion, so the 500mls EBL indication otherwise wouldn’t apply |  |  |  |
|  |  |  | Survey | TXA should not be used as a substitute for standard surgical haemostatic techniques, and the reasons for blood loss are multifactorial. I struggle to understand how a patient with normal fibrinolysis would benefit from TXA, and I can only think of a few indications e.g. diffuse bleeding from anti-platelet therapy or abnormal TEG in trauma where TXA would be helpful. Therefore, it seems unnecessary to routinely prescribe this agent. |  |  |  |
|  |  |  | Survey | The NICE guidance is currently focused on Estimated Blood Loss, (>500mls) is flawed. Firstly estimation of anticipated blood loss is inaccurate and unreliable, secondly actually recorded blood loss is an estimate. TXA has benefits over blood loss to reduce transfusion, it is felt that many surgeons feel it improves the surgical field and reduces post operative drain content and wound haematoma. |  |  |  |
|  |  | Education and training about using TXA | Interview | I think yes. I think there is enough communication and dissemination of the information (Pt12, Q38). | (n=18) | High |  |
|  |  |  | Interview | I would say I have not been trained in its utilisation at all. So then there was probably a slight absence of it, yeah. While I've never, I've never been aware of a single opportunity to have it discussed. So, I would imagine across the board (Pt7, Q21). |  |  |  |
|  |  |  | Survey | Availability of viscoelastic testing to diagnose hyperfibrinolysis and therefore not administer TXA blindly  Applicability of current evidence base to subspecialties and lack of sufficient evidence in these situations e.g. portal vein thrombosis with TXA administration  Concern regarding blindly giving TXA to the hypofibrinolytic phenotype |  |  |  |
|  |  |  | Survey | Not really - as an anaesthetist there is a large retrospective meta-analysis by the American Society of Anaesthesiologists that shows no significant risk of increased adverse cardio or cerebrovascular events, so feel comfortable with routine use. |  |  |  |
|  |  |  | Survey | Medical Evidence |  |  |  |
|  |  |  | Survey | No straightforward and evidence based |  |  |  |
|  |  |  | Survey | Colleagues widely unaware of guideline and anaesthetists concerned about (unproven) risks |  |  |  |
| **Opportunity** |  |  |  |  |  |  |  |
| **3. Environmental context and resources** | Material resources  (including availability and management), environmental impact including task environment | TXA is always available when I want to use it | Interview | Yeah. I haven't come across this situation where it hasn't been available to date (Pt16, Q44). | (n=20) | High |  |
|  |  | Inclusion of TXA on the WHO checklist | Interview | Yes, that is something that at the beginning it is discussed (Pt4, Q11). | (n=18) | High |  |
|  |  |  | Interview | I would give it even though I'm expecting less than 500mls because it is an easy-to-use drug, safe and is cheap (Pt1, Q3). |  |  |  |
|  |  |  | Interview | And I think that comes down, you know, that's a key question that I think sometimes gets lost in the lack of sensitivity of these checklists or this individual patient in front of you (Pt10, Q34). |  |  |  |
|  |  | TXA use based on the culture of the environment/team | Interview | So, it's now it's a question, I don't know if that's national or if it's just a Trust thing, but so it it gets mentioned on the WHO in other subspecialty areas. It’s alright. It's a culture thing. Um so. Like our orthopaedic surgeons are tuned into it, spinal surgeons are tuned into it. And other areas are less so, but they may not expect as regular blood loss. Just more irregular their blood loss. And then you got a few funny comments from other surgeons about it. And yes and no. If it's a relatively small operation plastics, then asking about TXA, it's a bit like. Yeah, no, you know, there it’s not relevant (Pt3, Q8). | (n=5) | Low |  |
|  |  |  | Survey | Hospital policy, Surgical perception of bleeding risk. |  |  |  |
| **4. Social / professional role and identity** | Professional identity and role. Identity within the professional group  Leadership. | Roles and guidelines for TXA within the team | Interview | So, our standard guidelines is if (they are) bleeding and has bled more than a litre than we should think about TXA (Pt8, Q26). | (n=20) | High |  |
|  |  |  | Interview | So, its inherent in the role I suppose. So in the morning meeting, we have a safety checklist, WHO checklist, and as part of that the anaesthetist for pretty much every case I do will be asked to give antibiotics and generally asked to give TXA. There aren’t any cases that I do that I can think of that I don’t give TXA (Pt15, Q43). |  |  |  |
|  |  |  | Interview | It's just like at the WHO briefing. We discuss what's the risk of blood loss over 500 and whether we should give tranexamic acid. I think the difficulty is judging accurately where the risks exceed the benefits and it would be nice to see where the benefits exceed the risks in those borderline cases where you don't expect blood lost over 500, but there's still a 10-20% chance it could happen. And is it worth giving the tranexamic acid then if the patient is also at high risk of DVT? I don't know the answer though (Pt9, Q29). |  |  |  |
|  |  |  | Interview | In the XXX hospital, we have a policy of greater than 500 mils of blood loss is predicted then tranexamic acid is given. The vascular surgeons are not that keen on trying tranexamic acid yet and I know they're looking into that. Because we have cell salvage for their cases, its not thought of as needed and we haven't had to transfuse any patients (Pt22, Q54). |  |  |  |
|  |  |  | Survey | Local guidelines. Presence of txa prompt on who checklist. Willing and familiar anaesthetists |  |  |  |
|  |  | Decision making is part of my role for TXA | Interview | We get asked generally on a case specific basis, whether we want to use TXA And in that situation, we pretty much the standard for those bigger cases would we'll it'll just be put through the WHO that we're going to use TXA and for the smaller cases that's then done on an ad-hoc basis on probably fairly nebulous decisions. Perhaps how, how difficult we think the surgery might be. Then the other form of bleeding risk. But really just on the difficulty of the surgery (Pt7, Q23). | (n=21) | High |  |
|  |  |  | Survey | Some surgeons believe it increases VTE (despite the evidence) and request that it is not given to all or specific patients, it is quite difficult to go against this |  |  |  |
| **Motivation** |  |  |  |  |  |  |  |
| **5. Beliefs about capabilities** | Self-efficacy, Control—of behaviour and  perceived competence  professional confidence  Empowerment  Self-esteem | Confidence to administer TXA to presurgical patients | Interview | I would say we're very confident (Pt6, Q17). | (n=21) | High |  |
|  |  |  | Interview | I think that now it has become more common for my colleagues to use TXA not only in spine surgery and because it is part of the protocol in knee and hip surgery, but also now it is widely used for head and neck surgery (Pt19, Q51). |  |  |  |
|  |  |  | Interview | I think we're all in the same boat. We are a little bit uncertain. They would say ‘yes, it's sensible to give it for a big liver resection. If someone's not had chemotherapy.’ Uh, before? No, it's obvious not (Pt9, Q27). |  |  |  |
|  |  |  | Interview | The anaesthetists feel very strongly. It shouldn't be given until the woman's haemorrhaged, and I agree with that because that's how it was used in the study (Pt11, Q36). |  |  |  |
|  |  |  | Survey | Yes. Because txa in elective surgery is often being used outside the terms of licensing there is pressure from pharmacy colleagues to only used if specific consent has been obtained pre operatively from the patient |  |  |  |
| **6. Beliefs about consequences** | Expected outcomes, evaluation/review, attitudes  consequences  Incentives/reward, perceived  risk/threat | TXA risks and benefits to surgical patients | Interview | I don't believe it has any risk and I think it's a good drug with benefits (Pt5, Q14). | (n=22) | High |  |
|  |  |  | Interview | So, I personally I'm not aware of a complication that we have attributed directly to TXA (Pt10, Q31). |  |  |  |
|  |  |  | Interview | I mean, I think for in the cardiac surgical setting, the benefits definitely outweigh the risks (Pt13, Q41). |  |  |  |
|  |  |  | Interview | I suppose the risk, well the risk profile in my mind, correctly or wrongly (laughs), is those patients who are at higher risk of thromboembolic events. There might be a risk in using it there (Pt2, Q6). |  |  |  |
|  |  |  | Interview | We’ve been using it for quite a while so you know how much difference does it make? I don’t really know. Because we always use it and therefore we don’t really notice whether it reduces blood loss or not. We just assume that it does (help) (Pt6, Q18). |  |  |  |
|  |  |  | Survey | Surgeons often underestimate potential EBL pre op. |  |  |  |
|  |  |  | Survey | Evidence is less clear in neuroanaesthesia & one of the potential side effects, seizures, is very relevant to our patient population |  |  |  |
|  |  |  | Survey | concerns regarding impact of TXA on microcirculation in microvascular anastomoses |  |  |  |
|  |  |  | Survey | As an anaesthetist, the surgical teams beliefs about benefits versus potential harms. I have noticed significant inter-specialty variation here. |  |  |  |
|  |  |  | Survey | surgical attitude, DVT risk |  |  |  |
|  |  |  | Survey | Usually influenced by surgeon preference in my experience. As an anaesthetist, I'm convinced by its evidence of efficacy and safety - we have more evidence on TXA when compared with all the other poor (or non) evidence-based interventions in peri-operative care and yet people remain sceptical of TXA. |  |  |  |
|  |  |  | Survey | A perception that it may increase thrombosis risk in the context of major vascular surgery with clamped vessels. |  |  |  |
|  |  |  | Survey | Surgeon Beliefs |  |  |  |
|  |  |  | Survey | Lack of knowledge of colleagues on using TXA |  |  |  |
|  |  |  | Survey | Largely emotional and cultural resistance from vascular surgeons (fear of thrombotic complications outweighs benefits of reduced blood loss) |  |  |  |
|  |  |  | Survey | I anaesthetise for renal transplant, where caution about renal vein thrombosis is an additional factor |  |  |  |
|  |  |  | Survey | In my experience the surgeons are more concerned regarding its adverse effects than any anaesthetist I have discussed with. Some surgical specialities are overly concerned about the potential adverse effects |  |  |  |
|  |  |  | Survey | Personal n FH clots Ca status and TEG Venous and arterial thrombosis risk Epilepsy risk Renal function |  |  |  |
|  |  |  | Survey | it is a clear fluid, easily mistaken for others.  It is fatal if administered intrathecally |  |  |  |
|  |  |  | Survey | There are also a large number of surgeons and anaes who are very resistant to being told what to do despite clear evidence. We would really benefit from knowing the risks to the baby are giving TXA before baby is out which is when it has been shown to work best. Women die from PPH and yet no-one will allow TXA to go on the obstetric WHO - it seems like madness. Some surgeons also want to assume that their surgery doesn't bleed.......in our hospital 1 or 2 clinicians are blocking it wholesale. then a few others follow and it just doesn't get through committee. |  |  |  |
|  |  |  | Survey | It is not a neutral drug. I prescribe and administer it routinely in Joint Replacement Surgery.  I obstetric practice nausea and hypotension are an issue. |  |  |  |
|  |  |  | Survey | I routinely use TXA, plus it is on our WHO checklist. However, it is clear that colleagues are less keen and trainees sometimes feel uncertain. My impression is this is mostly concern about risks and side effects, and less understanding about benefits. I operate on many patients with clotting disorders and work closely with haematologists, so I have more knowledge of benefits versus risks. |  |  |  |
| **7.Emotion** | How this makes you feel including stress, fear, anticipated regret, positive or negative | Feelings and concerns towards TXA including anticipated side effects and risks. | Interview | Nothing. It’s just like using local anaesthetic or antibiotics. It’s just something that you give (Pt6, Q19). | (n=22) | High |  |
|  |  |  | Interview | it feels like a very benign drug (Pt11, Q37). |  |  |  |
|  |  |  | Interview | …if it goes wrong, there is such a huge impact on the patients…So, what is the right thing in which situation? (Pt20, Q28). |  |  |  |
|  |  |  | Interview | And when I've heard the surgeons discuss it, it's fear rather than evidence. Or even anecdotes. Anecdotal experience (Pt10, Q32). |  |  |  |
|  |  |  | Interview | ‘I’ve known neurosurgeons to say no thanks cause of that worry. So, there’s still worrying about thrombosis (Pt3, Q33). |  |  |  |
|  |  |  | Interview | I think there's a there's a reluctance almost to give something that people may have this fear of causing thrombosis and graft failure in vascular. That's kind of that's almost an own goal for us (Pt18, Q49). |  |  |  |
|  |  |  | Interview | my main concern with the big cases is that the long cases, 6, 7, 8 hours of operating, meaning that you know, if I'm worried about increasing the risk of thrombosis...there is in general a hesitancy of using it...I think that it's a very safe drug and I think we should be using it more and more... I'm getting there to be convinced for, you know, regular use, so I'm using it more. But you know we are very, very, very scared of having thrombotic events in patients because they are all you know, having problems with mobility and things like that (Pt14, Q42). |  |  |  |
|  |  | Change in practice | Interview | I think no one is really worried about giving it. It is just the more that they are not familiar and have not have never used it before in their twenty year long career, so they, they feel reluctant of suddenly doing something new (Pt17, Q46). | (n=5) | Low |  |
|  |  |  |  |  |  |  |  |
| **8. Motivation and goals** | Intention and stability of intention. Goals,  Motivation and commitment | A national measure for improvement may affect TXA usage | Interview | I guess so. People tend to fall into being users or not seem to bother with it. All the surgeons I work with are very keen on it (Pt1, Q4). | (n=6) | Low |  |
|  |  |  | Interview | I mean we all know that if you attach a financial benefit to doing something in any trust that's switched on, well we will endeavour to comply (Pt6, Q20). |  |  |  |
|  |  |  | Interview | It's probably not because I think it probably is being used appropriately and in the correct way and at the correct time in the majority of cases only because we're almost, as I said, too trigger happy with TXA now (Pt8, Q25). |  |  |  |
|  |  |  | Interview | It can be…The problem…is the teams actually doing the work don't get the benefit (Pt7, Q22). |  |  |  |
|  |  | Education and training regarding risk factors would support usage of TXA | Interview | So maybe having some education and particularly evidence and potential risk factors would help (Pt16, Q45). | (n=18) | High |  |
|  |  |  | Interview | Well, I think even I, even though I blood loss is minimal, I mean if it's 200 and you can bring it down to 100 and I think it's still of value (Pt17, Q47). |  |  |  |
|  |  |  | Interview | You know, having something that could potentially, you know, reduce the amount of bleeding is something useful (Pt14, Q2). |  |  |  |
|  |  | Reminding people about its availability and effects can help with the uptake of TXA | Interview | I think reminding everybody that is available, what it does. You know it's all very well. Almost like you should revisit the original Canadian transfusion study you know. The more times you reiterate it, to the more people then they would know about it (Pt21, Q53). | (n=6) | Low |  |
|  |  | Additional evidence would affect usage of TXA | Interview | I think there needs to be; there is likely to be a period of five or ten years. I suspect where it's true role is actually found. And as I've said, you know most of the tranexamic acid I'm giving, I think is in non-evidence domains and I'm not entirely sure that people are actually genuinely trying to find the evidence for it's benefit. Or for its potential harms in those contexts (Pt10, Q35). | (n=10) | Medium |  |
|  |  |  |  |  |  |  |  |
| **Others** |  |  |  |  |  |  |  |
| **9. Skills** | Skills including competence, ability and practical skills development | The clarity of guidelines affects my use of TXA | Interview | So our standard guidelines is if (they are) bleeding and has bled more than a litre than we should think about TXA…But it's usually, you know, because it's such a standard part of our protocol, it's just usually ‘oh, (they are) now bleeding. Please, can you give some TXA, please?' (Pt8, Q24). | (n=20) | High |  |
|  |  |  | Interview | There will be stuff in the XXXX that I have looked at and forgotten. Uh, yeah. But again, that's all anaesthetic stuff. And but I presume the evidence is there. I've not looked at it (Pt3, Q9). |  |  |  |
|  |  |  | Interview | I think that some speciality's would be good to have some more evidence in those specific group of patients because studies that are very focused on trauma patients, there's a lot of evidence with trauma patients, but maybe not much evidence on vascular patients. Although there's some studies approach vascular patients and cardiac patients all of that population group. I think that some specialities have more when more evidence like others. For example, oncology patients, it’s a very specific group and population with their characteristics, that more evidence would be useful (Pt12, Q39). |  |  |  |
|  |  | Experience affects my decision making in using TXA | Interview | I think I'm probably influenced by my experience and my previous kind of working with teams where I think you know, there's a dogma is a bit of a kind of influence, influential word (Pt18, Q50). | (n=19) | High |  |
|  |  |  |  |  |  |  |  |
| **10. Nature of the behaviour** | Direct experience/past behaviour, routine  And representation of tasks | It is used widely and as part of the routine | Interview | And I think its just the sheer number of cases we have used it on without incident or problem (Pt5, Q16). | (n=11) | Medium |  |
|  |  |  | Interview | the TXA question became part of the of the WHO procedure. So that made everyone realize, well, apparently for all the specialties it is a completely normal thing to include in your daily practice (Pt17, Q48). |  |  |  |
|  |  |  | Interview | I think now it's part of our WHO checklist and it's asked every single time, every procedure and we’re not entirely sure where this came from because I don't think it was actually the vascular surgeon involved in this. But it seems to be the Trust, you know, the WHO checklist we do at the beginning of every operation that seems to have changed…and suddenly there. ‘Do you want TXA as part of every question?’ Every time we discuss it and majority of us say no, you know (Pt20, Q52). |  |  |  |
|  |  |  |  |  |  |  |  |
| **11. Social influences** | Social support and group norms  Team working, culture and group conformity.  Learning and modelling | Recent blood shortages brought about an increase of the use of TXA | Interview | Well, now you're having the surgeons worried about their blood product usage has certainly focused their minds about TXA usage. We have regular audits and cardiac and orthopaedic surgeons are looking more and more blood product usage as a surrogate for quality of care (Pt1, Q5). | (n=5) | Low |  |
|  |  |  | Interview | Yes, in terms of saving how much blood is given to the patient, TXA if given where indicated can have a good impact as well (Pt4, Q12). |  |  |  |
|  |  |  | Interview | I would say that the fact that there was a blood shortage was also a driver for the increased use of TXA as with any other patient blood management techniques I would say as well. So yes yes I think it's trying to find strategies to mitigate the short age of blood (Pt12, Q40). |  |  |  |
|  |  |  | Survey | Blood shortages have increased use in our institution. |  |  |  |
|  |  | Changes in practice with TXA take time | Interview | When I started, nobody gave it. So, I started doing anaesthetics in 2000. So, nobody gave it back then. And then when it was a trainee. And it used to be cardiac only (Pt3, Q10). | (n=6) | Low |  |
|  |  | Who has direct responsibility for TXA decision making | Interview | I think it’s the consultant surgeon (Pt2, Q7). | (n=10) | Medium |  |
|  |  |  | Interview | The final decision will be from the surgeon or anaesthetic team, but it will mostly be the anaesthetist that gives it (Pt4, Q13). |  |  |  |
|  |  | Social influence of TXA | Survey | Often culture within different surgical specialties |  |  |  |

*Summary of domains from Atkins, L., Francis, J., Islam, R. et al. A guide to using the Theoretical Domains Framework of behaviour change to investigate implementation problems. Implementation Sci 12, 77 (2017). https://doi.org/10.1186/s13012-017-0605-9
